# Potential Virus Involvement in Alzheimer’s Disease: Results from a Phase IIa Trial Evaluating Apovir, an Antiviral Drug Combination

**DOI:** 10.3233/ADR-210301

**Published:** 2021-05-28

**Authors:** Nina Lindblom, Lars Lindquist, Jacob Westman, Mikael Åström, Roger Bullock, Suzanne Hendrix, Lars-Olof Wahlund

**Affiliations:** a Apodemus AB, Solna, Sweden; b Clinic for Infectious Diseases and Institution of Medicine, Karolinska University Hospital and Karolinska Institutet, Huddinge, Sweden; c Roger Bullock Consulting Ltd, Swindon, UK; d Pentara Corporation, Salt Lake City, UT, USA; e NVS Department, Section of Clinical Geriatrics, Karolinska Institutet and Karolinska University Hospital, Huddinge, Sweden

**Keywords:** Alzheimer’s disease, amyloid-β, antiviral agents, clinical trial, infection, pleconaril, ribavirin

## Abstract

**Background:**

Accumulating data suggest infectious agents are involved in Alzheimer’s disease (AD). The two primary aims of this trial were to assess safety and efficacy of an antiviral drug combination on AD progression.

**Objective:**

The trial evaluated whether Apovir, a combination of two antiviral agents, pleconaril (active on enteroviruses) and ribavirin (active on several viruses), could slow AD progression.

**Methods:**

Sixty-nine patients 60–85 years were treated with Apovir or placebo for 9 months and followed until 12 months after end of treatment. Cognitive tests, safety, biomarkers, drug plasma, and cerebrospinal fluid concentrations were assessed.

**Results:**

The tolerability of Apovir was compromised as demonstrated by the large drop-out rate and increased frequency and severity of adverse events. The primary endpoint, demonstrating a difference in change from baseline to 9 months between groups in ADAS-cog total score, was not met (*p* = 0.1809). However, there were observations indicating potential effects on both ADAS-cog and CDR-SB but these effects need to be verified. Also, there was a decrease in cerebrospinal fluid amyloid-β in Apovir at 9 months (*p* = 0.0330) but no change in placebo.

**Conclusion:**

This was the first randomized, placebo controlled clinical trial exploring antiviral treatment on AD progression. The trial is considered inconclusive due to the large drop-out rate. New trials are needed to verify if the indications of effect observed can be confirmed and which component(s) in Apovir contributed to such effects. Pleconaril alone may be studied to improve the tolerability and to verify if enterovirus is involved in the disease process.

## INTRODUCTION

Alzheimer’s disease (AD) is the most common dementia disorder and contributes to most of all dementia patients [1]. It has a major impact on society as well on the people with the disease and their families. It has been estimated that the worldwide cost of dementia will reach US$ 1 trillion in 2018 [2], and that the number of persons affected will double approximately every 20 years [3]. Despite decades of major efforts to delineate the etiology of AD and to develop effective treatments for the disease, there is still no cure for AD.

It is generally accepted that several etiological factors including genetic, lifestyle, and environmental factors are involved in AD. Further it is generally agreed that the contribution of such various factors may differ between subjects [4]. On a molecular level, AD is characterized by presence of brain amyloid-β (Aβ) neuritic plaques, hyperphosphorylated tau (p-Tau) neurofibrillary tangles, and associated neuroinflammatory processes. The underlying molecular mechanisms driving these processes are not fully understood. Aβ has been the primary target in drug development in AD modifying treatments during the last decades, where it has generally been targeted as a toxic and disease-causing agent. However, Aβ has been found in all vertebrates studied to date and the high degree of molecular conservation between species indicates that Aβ is important for species survival [5]. Further, *in vitro* and *in vivo* studies suggest that Aβ has important physiological functions in the body including protecting the body from infections, repairing leaks in the blood-brain barrier, promoting recovery from injury, and regulating synaptic function [5]. Potentially, the factors driving an increased Aβ production would therefore be a better target for AD treatment than the Aβ itself. One such factor linking increased Aβ and AD is infections. Cells affected by infectious agents have been shown to produce increased amounts of Aβ [6–8], and Aβ has been demonstrated to possess antimicrobial effects [9–11]. It has been suggested that Aβ has neuroprotective effects under certain physiological situations and neurodegenerative effects under other conditions [12], e.g., during aging or increased infectious pressure. Infectious agents may be involved in AD directly, but it has also been suggested that an altered immune and inflammatory response to infectious agents may be important in the development and progression of AD [13, 14].

The first to suggest microorganisms to be involved in AD pathology was Oskar Fischer who together with Alois Alzheimer argued that senile plaques could be formed by microorganisms. Much later, in the mid 1970s, it was suggested that infectious agents could be involved in AD [15, 16] and several infectious agents including bacteria, fungi, and viruses [6, 17–25] have been suggested to be involved in the development and/or progression of AD.

Evidence suggests that pathogen induced inflammation and accumulation of Aβ may contribute to AD [7]. Several studies have reported finding a higher proportion of patients with infectious agents in postmortem brains of AD patients compared to controls [17, 26, 27]. Additional evidence pointing to the role of infections in AD includes data on infectious burden where the presence of antibodies against a larger number of infectious agents was shown to be associated with an increased risk for AD [28]. It has also been shown, in a Canadian study, that vaccination against diphtheria, tetanus, poliomyelitis, and influenza lowered the risk of AD [29]. Further, periodontal bacterial infection [30] and gut microbiota have been implicated in AD [31]. Several mechanisms could explain a potential infectious contribution to AD including; persistent low grade infections; repeated/reactivation of one or several infectious agents; and a high burden of central and/or systemic infections. These effects could either be direct or could be mediated via alterations in the immune response that is also known to occur during aging. Herpes virus (HSV) is the infectious agent with most published data linked to AD. For example, publications have shown more HSV in the brains of AD patients compared to controls [27]. An animal study with recurrent HSV-1 infections in mice was able to demonstrate that thermal stress-induced virus reactivations lead to accumulation of several AD hallmarks including Aβ, p-Tau, and neuroinflammation markers and that these correlated with cognitive deficits in mice [32]. Very interestingly, a retrospective population-based study from Taiwan has recently shown that HSV infected patients more than doubled the risk of developing dementia and further that treatment of HSV infections with anti-herpes medication reduced the risk of developing dementias of various forms including AD [33]. Currently, we are aware of two clinical trials evaluating the effect of the herpes medication valacyclovir on AD (NCT03282916 and NCT02997982). Also, chronic periodontitis where the bacteria *Porphyromonas gingivalis* is a keystone pathogen [34] has been identified as a significant risk factor in AD [35] and it has shown that chronic oral application of the *Porphyromonas gingivalis* in mice lead to the bacteria being found in the brains of the mice along with increased expression of inflammatory markers TNFα, IL-6, and IL-1β and associated cognitive deficits [30]. *Porphyromonas gingivalis* DNA and the antigen of its toxic protease, gingipain, have been found in AD brains [36]. A phase II/III clinical trial evaluating the effect of COR388 (NCT03823404), a substance inhibiting gingipain, is now being conducted.

Enterovirus (EV) is a large virus group consisting of more than 200 human pathogenic viruses including, e.g., polioviruses that causes poliomyelitis and rhinoviruses that causes the common cold. In general, EV infections are mild or even asymptomatic but they may also become serious or life threatening, especially in subjects with a poor immune system or with certain underlying medical conditions. Some EV strains have high tropism for the central nervous system (CNS) and neurological complications from EV infections include, e.g., aseptic meningitis, encephalitis, and acute flaccid paralysis. It has been demonstrated that EVs can access the CNS via several routes and further they can evade the immune system and cause persistent infections [37] similar to other infectious agents implicated in AD, e.g., HSV and *Borrelia burgdorferi*.

Picornavirus, the virus family to which EV belongs, has been pointed out as the most commonly encountered infectious agent in mankind [38] and EV which is the largest group of human pathogenic viruses and cause on average 2-3 infections in adults each year [39]. There are currently no publications directly supporting the involvement of EV in the development or progression of AD although a few case reports on dementia and EV have been published [40, 41]. Also, there are some publications linking EV with other neurodegenerative diseases [37, 42]. However, several reports have also failed to establish a link between EV and AD [27, 43, 44]. This could however be due to low levels of virus in combination with the methodological analysis protocols used and time-point of sampling [45, 46]. Indeed the EV surveillance guideline points out that a cell culture step before analysis is done by many EV laboratories [47], and a recently published study protocol for detecting low numbers of EV in both tissues and body fluids included a cell culture step in EV susceptible cells or pre-PCR before analysis [48]. Therefore, we conclude that studies not optimized to identify EV do not provide evidence against the involvement of EV in AD. Further, EV is a common etiological factor of meningitis [49] and encephalitis in adults. One study reported EV in 51.6%of the cases whereas bacterial meningitis was found in 14.1%and HSV in 8.2%[50]. It has also been shown that several EV can persist in the CNS and can trigger apoptosis and autophagy which may contribute to infection associated long-term sequalae such as neuropathogenesis [51]. Together these observations argue for EV as an interesting target to explore if central infections in general are believed to be involved in AD. At the time of initiating this trial we also had unpublished, potentially biased data from five patients treated with pleconaril in combination with ribavirin (i.e., the Apovir drug combination) and/or efavirenz for up to three years. These data supported the decision to start the trial as well as decisions around the trial design. However, due to later discovered issues with the reliability of these results data they are not further presented or discussed. We believe that common infections including EV might be involved in the pathogenesis of AD and that treating chronic infections or preventing future infections by means of antiviral therapy may reduce the symptoms of the disease and/or slow the rate of disease progression. The trial hypothesis was to evaluate the safety and efficacy of the antiviral drug combination Apovir on AD progression.

Ribavirin is a low-molecular weight nucleoside analog and inosine monophosphate dehydrogenase inhibitor, a broad-spectrum antiviral drug with activity against a variety of RNA and DNA viruses. It is approved for treatment of hepatitis C in combination with interferon, and for respiratory syncytial virus (RSV) in children. Ribavirin is known to potentiate the antiviral effect of interferon considerably while being only modestly effective against hepatitis C on its own [52, 53]. In addition, although mixed opinions exists in the literature there are publications showing that ribavirin is effective against HSV both on its own [54] and in combination with acyclovir where it has been shown to potentiate the effect of acyclovir [55]. Activity of ribavirin against EV has been demonstrated *in vitro* [56–58], *in vivo*, and clinically in EV induced mild hand-foot-mouth disease [59, 60]. Several of the viruses on which ribavirin is effective—HSV, RSV, and hepatitis C—have also been implicated in AD [54, 61, 62]. The mechanism of action for ribavirin is not fully understood and the prevailing assumption is that its antiviral activity is exerted directly through lethal mutagenesis of the viral genetic material [63]. It has, however, also been claimed that the overwhelming success of ribavirin largely derives from its excellent performance in synergy with interferon based therapies which has been extended to combination treatments with direct acting antivirals where several lines of evidence pointed to a concomitant immunomodulatory effect [64].

The second antiviral drug, pleconaril, also known as APO-P001 or Picovir, is a direct-acting antiviral primarily active against the rhinovirus subgroup of EV. Pleconaril inhibits the virus replication by binding to the virus capsid preventing the virus from exposing its RNA and from host cell attachment. Pleconaril has previously undergone clinical development for treatment of the common cold. Between 1996 and 2005, more than 4,500 subjects were exposed to it in different clinical trials [65–68] where pleconaril was also shown to slightly shorten the duration of meningitis symptoms [69, 70]. Additionally, more than 300 subjects have been treated with pleconaril in a compassionate use program designed to treat patients with severe, disabling and/or life-threatening presumptive enteroviral syndromes [49, 65, 71–73].

The reason for selecting the studied drug combination was primarily to assess if EV is involved in AD, and to do that, pleconaril was selected. Ribavirin was added to the treatment because it has been shown to have some effect on EV, to potentiate antiviral effects and because generally, antiviral combinations are preferred over monotherapy; this is partly based on that theoretically, an antiviral combination decrease the risk for development of viral resistance. Although pleconaril is EV selective ribavirin is active against many viruses (but with very low potency at the doses studied) and also has immunomodulatory effects [64]. Therefore, a possible effect following treatment with this drug combination would indicate an antiviral effect in AD primarily from EV, but it would not be possible to exclude that ribavirin had contributed to the effect by acting on other viruses or via its immunomodulatory effects (these are beyond the scope of this publication even though it could be relevant for AD).

Presently, there is no available treatment that can stop or reverse the progression of AD. A large majority of the drug development projects aiming to change the cause of AD progression have targeted a limited number of treatment principles whereof the most common target has been Aβ, yet there have only been hints of success. During the last couple of years several large, late phase AD clinical trials evaluating BACE inhibitors, Aβ antibodies, and 5-HT6 antagonists have failed. There is an urgent need to broaden the development pipeline in AD research and explore alternative hypotheses in the search of new and effective treatments for AD. Antiviral treatment is one such approach and to our knowledge this was the first placebo-controlled trial evaluating the effect of antiviral treatments in AD. The aim of the trial was to investigate the safety and efficacy of Apovir, on the progression of AD during 9 months treatment and 12 months post treatment follow-up.

## MATERIALS AND METHODS

### Trial design

This was a single site, randomized, double blind, placebo-controlled, parallel-group trial evaluating the effect of Apovir, a combination of two antiviral agents, pleconaril and ribavirin, on patients with AD. Patients were randomly assigned (1:1) to Apovir or placebo. The trial consisted of two parts: *the original part of the trial* including a screening period, a 9-month treatment period, and a 1-month follow-up period; and the *follow-up part of the trial* consisting of two follow-up visits at 6 and 12 months after end of treatment. The follow-up part of the trial was added to the trial protocol by an amendment before the first patient had reached the 6-month follow-up visit. The follow-up part of the trial was added to assess whether a potential treatment effect would be sustained after treatment had been discontinued.

The initial sample size was 60 patients; it was based on assuming a common standard deviation of 6.7, a difference in means of 5.2 points for the Alzheimer’s disease assessment scale - cognitive (ADAS-cog) score at 9 months, and an 80%power to detect a difference using a two sample *t*-test with a two-sided significance level of α= 0.05 (a sample size of 28 patients per group). Based on the interim analysis, when 30 patients had conducted 6 months, the trial could be stopped for futility or the sample size could be increased to enroll an additional 30 patients within the protocol. Patients who dropped out during the recruitment period could be replaced.

### Drugs, doses, and administration

Pleconaril, ribavirin, and their respective placebos were administered as 200 mg oral capsules. Patients received written instructions for dosing. Initially, one capsule of the respective drug was to be taken in the morning and two in the evening; however, after the dose reduction of ribavirin one capsule was to be taken in the morning and evening. Capsules were to be taken with food, preferably with high fat to improve the Apovir exposure and to reduce the risk of adverse events (AEs).

Pleconaril (3-[3,5-dimethyl-4-3-(3-methyl-1,2-oxazol-5-yl)propoxy]phenyl]-5-(trifluoromethyl)-1,2,4-oxadiazole) was prepared through a four-step synthetic process starting from 3,5-dimethyl isoxazole. The synthesis was performed by Anthem BioSciences Ltd in Bangalore, India under GMP in a 40 kg scale following the same synthetic route as described in Diana et al. (US 5464848). The active pharmaceutical ingredient (API) was formulated in hard gelatin capsules size 0 using pregelatinized starch as the major excipient. The ribavirin (1-[(2R,3R,4S,5R)-3,4-dihydroxy-5-(hydroxymethyl) oxolan-2-yl]-1H-1,2,4-triazole-3-carboxamide) drug product (hard gelatin capsules, size 0) was an overencapsulated copegus tablet (Copegus® 200 mg tablets, purchased from Roche AB, Box 47327, 100 74 Stockholm, MA number: 18614) with pregalatinized starch as the filler. Both drug products were produced by Apotek Produktion & Laboratorium AB, in Stockholm, Sweden

The dose selection of ribavirin for the present trial (600 mg/day) was clearly lower than the commonly used dose of 1000–1200 mg/day. The selected dose was chosen with the aim to prevent the frail older patient population under study from the well described side effects commonly observed during treatment with ribavirin. However, compromised tolerability was observed during the trial (see Results), and although the Data Safety Monitoring Board (DSMB) did not determine that a dose reduction was needed, the sponsor decided to reduce the dose of ribavirin for all patients from 600 mg/day to 400 mg/day aiming to improve the tolerability of the treatment. The dose reduction was implemented when 39 patients had been randomized, about 3.5 months after the first patient was randomized. Subsequently randomized patients were treated with 400 mg/day from the start. The protocol was also amended to describe that ribavirin treatment could be discontinued for tolerability reasons and that the treatment could continue with pleconaril alone. The selected dose of pleconaril, 600 mg/day, was lower than the 800–1200 mg/day doses previously reported to have been used. The rational for selecting a lower dose was again based on treating an old and frail population. In addition, treatment periods with pleconaril longer than 6 weeks had not previously been reported. Further, the sponsor had data on a number of patients who had received high doses of pleconaril in combination with ribavirin and/or efavirenz over treatment periods up to 3 years on a named patient treatment basis. Out of these patients, three patients diagnosed with amyotrophic lateral sclerosis had encountered deep venous thrombosis possibly linked to pleconaril. This information also contributed to the decision to, choose a lower dose of pleconaril. Drug plasma concentrations were measured in the trial to confirm if they were within expected range based on previous reports and not altered due to the combination treatment. Further, ribavirin plasma concentrations were routinely assessed by the DSMB during the trial to ensure levels were acceptable. The DSMB had the authority to reduce the dose/withdraw ribavirin if needed based on plasma concentration level for a concerned patient during the trial; this, however, was not needed.

### Subjects

Eligible patients were male and female patients aged 60–85 with an existing diagnosis of AD coded according to the 10th revision of the International Statistical Classification of Diseases and Related Health Problems (ICD-10) criteria and a Mini-Mental State Examination (MMSE) score between 21 and 27. Patients were thus not diagnosed within the frames of the trial. However, imaging reports generated in relation to diagnosis were assessed by the investigators before inclusion and subjects with signs of cerebrovascular changes, large brain infarctions, large areas of white mater changes as well as brain tumors, previous bleedings and brain trauma were excluded. Further medical history as well as screening laboratory data were evaluated to exclude patients that did not meet the criteria for participation in the trial. Subjects receiving symptomatic treatment for AD (acetylcholinesterase inhibitor or memantine) were required to have been on stable treatment (drug and dose) for at least 3 months prior to inclusion. Patients also had to have a relative or caregiver who could participate at the patient’s visits and help with the medication during the trial. Patients were not allowed to have HIV or active hepatitis B or C. Patients with a serious cardiac disease, impaired kidney function, and patients who had undergone a major surgical procedure within 4 weeks prior to inclusion were not allowed to participate in the trial. Although concurrent AD treatment was not required, all patients randomized were on stable treatment with acetylcholinesterase inhibitor/memantine and treatment was to remain unchanged during the original part of the trial. Apolipoprotein E4 status was not assessed. Presence of EV infection was not assessed by either measuring virus titers or antibodies to EV.

### Recruitment and pre-screening

The trial was conducted between November 2013 and June 2016. Patients were recruited via advertising in local newspapers, flyers, and postings at patient organizations’ webpages. Further, medical record searches conducted by memory clinics identified potentially eligible patients. Patients were subsequently contacted by personnel at the patient’s memory clinic who asked the patient whether he/she would like to be contacted by the clinical trial site personnel for additional information and a possible participation in the trial. In total, six memory clinics (see Acknowledgments) in the Stockholm area participated in identifying patients for the trial. Potentially eligible patients were subsequently prescreened by the clinical trial site and scheduled for a screening visit if they fulfilled the pre-screening criteria. At the screening visit, patients and their caregivers/relatives were informed about the trial and signed an informed consent form before any study related procedures took place. Presence of an AD diagnosis was required for participation in the trial and was confirmed from the patients’ medical records. This was made possible by the common electronic journal system held by the Stockholm’s Läns Landsting to which site personnel could log in and review patient information after having received approvals from the patients, in accordance with the ethics approval.

### Assessments

Patient demographics and baseline characteristics including age, sex, ethnic origin, weight, height, medical history, concurrent diseases, and prior and concomitant medications were assessed.

#### Efficacy assessments

Four different tests were used to assess cognitive function during the trial: MMSE [74] and Clinical Dementia Rating –sum of boxes (CDR-SB) [75], which were administered by the investigator; and the ADAS-cog 11 item [76] and Alzheimer Quick Test (AQT) [77] which were administered by trained study nurses. ADAS-cog was administered during both the original and long-term follow-up parts of the trial whereas MMSE, CDR-SB, and AQT were administered during the original part of the trial only. During the trial, the administration of AQT was changed, for this reason the AQT results are considered unreliable and will not be presented.

Trough concentrations of pleconaril and ribavirin in plasma and cerebrospinal fluid were measured during the original part of the trial. Laboratory analyses were performed by Karolinska Universitets Laboratoriet.

#### Safety assessment

Safety was assessed throughout the original part of the trial and until 1 month after end of treatment. Safety assessments included frequency and severity of AEs, vital signs, physical examination, and laboratory evaluations of clinical chemistry and hematology variables. Signs of muscle inflammation (clinical signs, LDH and CK) and signs of thrombosis (clinical signs, d-dimer and CRP) were also assessed. EEG was assessed at screening and after 1- and 9-months treatment. A DSMB reviewed safety data regularly during the original part of the trial. Laboratory analyses were performed by Karolinska Universitets Laboratoriet. AEs were coded according to MedDRA and grouped by system organ class and preferred term. The investigators assessed AEs by severity (mild, moderate, severe), seriousness (serious, non-serious), and relatedness (unlikely, possible, probable).

#### Pharmacodynamic assessments

Cerebrospinal fluid (CSF) samples were collected at baseline and at 9 months to exploratory assessment of the pharmacodynamics biomarkers Aβ_42_, tau, and p-Tau. Analyses were performed by Karolinska Universitets Laboratoriet.

As an exploratory analysis the correlation between plasma drug concentrations and change in ADAS-cog was calculated. The change from baseline in CSF biomarkers Aβ_42_, tau, and p-Tau were also assessed as exploratory.

### Data collection

All data collected in the trial was recorded in the trial electronic database. The electronic case report form (eCRF) Viedoc was used for data collection.

### Statistical analyses

An interim analysis was conducted when 30 patients had completed the 6-month visit. The purpose of the interim analysis was to re-estimate the sample size and, if needed, increase the number of patients in the trial.

The main analysis of the primary efficacy variable, ADAS-cog total score at 9-months post baseline, was to be performed on the Full analysis set (FAS) using a mixed-effects model for repeated measures (MMRM) if data met the assumptions for the model. The data set was judged not to fulfill the assumptions for the MMRM analysis with regards to error terms following normal distribution. Therefore, the following non-parametric alternative analysis on observed cases was performed:

The primary efficacy endpoint was change from baseline until 9 months post baseline with no imputations of missing data, i.e., the analysis is an observed cases analysis. A supportive sensitivity analysis using the per protocol analysis set (PPAS) was also performed using the same statistical methods. The hypothesis that the change in ADAS-cog is equal among patients randomized to placebo and patients randomized to Apovir was tested with the exact (i.e., not the normal approximation of the test) Wilcoxon rank sum test. The 2-sided *p*-value is presented for all tests and is considered statistically significant if it is less than 0.0500.

**Fig. 2:**
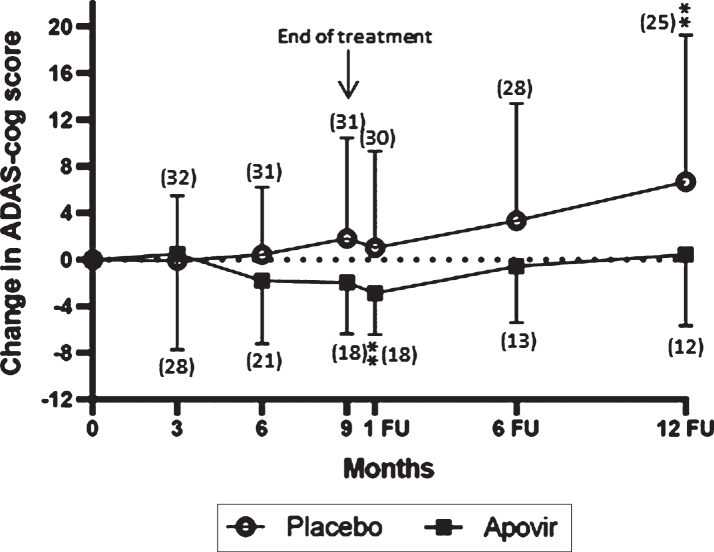
Change in ADAS-cog 11 from baseline over a 9-month treatment period and a 12-month follow-up period in the Full analysis set. Vertical bars represent the standard deviation. **represents significant within group change < 0.01 compared to baseline. Numbers within brackets represent number of patients per group at respective time-points. Analyses are based on observed cases are impacted by drop-out biases and should be interpreted cautiously.

The statistical analysis plan described that ADAS-cog results should be omitted for a concerned patient visit if the items “word recall” and/or “word recognition” were not fully completed. During the 6- and 12-month follow-up visits, 6 patient visits did not fulfill this criterion; however, this was due to disease progression, so the patient scores were included in the analysis for ADAS-cog in Fig. 2, Table 3, and Table 4 as we deemed this to be more correct.

**Table 3:** ADAS-cog change from baseline during the original and follow-up parts of the trial, Full analysis set

ADAS -cog score	Apovir	Placebo	Between group *p*
Change from baseline to 6 months
n/N	21/29	31/33
Mean (SD)	–1.810 (5.403)	0.430 (5.798)	0.1120
Within group *p*-value	0.0878	0.9520
Change from baseline to 9 months
n	18/29	31/33
Mean (SD)	–1.963 (4.398)	1.817 (8.623)	0.1809
Within group *p*-value FAS	0.1011	0.9619
Change from baseline to 1-month Follow-up
n	16/29	30/33
Mean (SD)	–2.876 (3.551)	1.012 (8.291)	0.0545
Within group *p*-value	0.0082	0.5734
Change from baseline to 6-month Follow-up
n	13/29	28/33
Mean (SD)	–0.589 (4.821)	3.334 (10.074)	0.1975
Within group *p*-value	0.4871	0.3832
Change from baseline to 12-month Follow-up
n	12/29	25/33
Mean (SD)	0.417 (6.082)	6.680 (12.596)	0.0700
Within group *p*-value	0.9160	0.0063

Analyses based on observed cases are impacted by drop-out biases and should be interpreted cautiously. Wilcoxon’s signed rank test and Wilcoxon’s rank sum test used to assess within and between group changes respectively. ADAS-cog, Alzheimer’s Disease Assessment Scale - cognitive.

**Table 4:** Clinically relevant change, change in ADAS-cog score from baseline, observed cases, Full analysis set

	6 Months		9 Months		1-Month Follow-up		6-Month Follow-up		12-Month Follow-up	
	Improve	Worsen	Improve	Worsen	Improve	Worsen	Improve	Worsen	Improve	Worsen
Placebo	5 (16.1%)	6 (19.3%)	6 (19.3%)	7 (22.6%)	6 (20.0%)	6 (20.0%)	4 (14.3%)	5 (17.9%)	2 (7.1%)	8 (28.6%)
n	31		31		30		28		25
Apovir	8 (38.1%)	3 (14.3%)	6 (33.3%)	1 (5.6%)	8 (50%)	1 (6.3%)	4 (30.8%)	2 (15.4%)	4 (30.7%)	2 (15.4%)
n	21		18		16		13		13
FAS/ITT	*p* = 0.1489	*p* = 0.1318	*p* = 0.0328	*p* = 0.1309	*p* = 0.2026

Analyses based on observed cases are impacted by drop-out biases and should be interpreted cautiously.

Although the preplanned primary MMRM analysis requires the assumption of error terms following a normal distribution, the alternative non-parametric analysis performed of observed cases requires an assumption of missing completely at random. Because the MMRM is robust to the normality assumption, it requires a less strict assumption of missing at random and a *post hoc* MMRM analysis was also conducted. Secondary efficacy endpoints, safety, pharmacokinetic, and exploratory variables and endpoints were to be presented with descriptive statistics. Several *post hoc* analyses were conducted to:

1. Assess within and between group changes at various time-points using 2-sided Wilcoxon signed rank test and Wilcoxon rank sum test respectively for ADAS-cog and CDR-SB.
2. Evaluate the association between investigational medicinal products (IMP) plasma concentrations and change in ADAS-cog from baseline to 9 months using Pearson correlation coefficient.
3. Assess change in biomarkers between and within groups from baseline to end of treatment using *t*-tests.
4. Account for the large drop-out and missing data in the Apovir group with the following approaches:

• Replacing missing data with a z-score imputation (iterated until convergence). The z-score imputation is calculated by using the z-score of the patient at the last observed visit and carrying the value forward to each of the later visits using the same z-score relative to the mean and SD of the later visits (iteration 1). After imputing the values for the later visits, the mean and SD of those later visits are re-calculated including the imputed z-score data. These new means and SDs are then used for imputing the values (iteration 2). This iterative process is repeated until the means do not change with the inclusion of the imputed values.
• Assessing the effect on ADAS-cog and CDR-SB for patients in the PPAS versus the FAS using 2-sided Wilcoxon rank sum test.
• ADCOMS [78] is a weighted linear combination of items from the ADAS-cog, MMSE, and the CDR-SB and was also assessed. *ADCOMS* is calculated as: ADCOMS = ADAS-cog Delayed word recall^ *^0.008 + ADAS-cog Orientation^ *^0.017 + ADAS-cog Word recognition^ *^0.004 + ADAS-cog Word finding difficulty^ *^0.016 + MMSE Orientation time^ *^0.042 + MMSE Drawing^ *^0.038 + CDR-SB Personal care^ *^0.054 + CDR-SB Community affairs^ *^0.109 + CDR-SB Home and hobbies^ *^0.089 + CDR-SB Judgement and problem solving^ *^0.069 + CDR-SB Memory^ *^ 0.059 + CDR-SB Orientation^ *^0.078.
• Assessing the effect size estimates on original data and z-score imputed data after removing matched ranked placebo patients for Apovir patients who dropped out. The patients were ranked separately within each treatment group at baseline and were matched 1 : 1 with the same ranked patient from the other group. Patients who dropped out of the Apovir group then had their matched placebo patient dropped from the analysis. Effect at 9 months were assessed for ADAS-cog, the composite score ADCOMS [78], CDR-SB, and MMSE. The *effect size* is the percentage of the placebo group decline that is reduced by active treatment, so a 100%effect size means there is no decline in the active group compared to a progressive decline in the placebo group. A 50%effect size would be a slowing of 50%of the placebo decline.
• Removing worst placebo patients at baseline (highest) and worst responders (largest increase) in ADAS-cog at 9 months to simulate the drop-out if the worst patient had dropped out of the Apovir group.
• An MMRM analysis was performed using change from baseline for each outcome variable as the response, with terms in the model for visit as a categorical variable, baseline score and age as covariates, treatment group, treatment group by visit interaction and baseline by visit interaction. Least squares means from the model for each treatment group were used as the estimates and treatment groups were compared at each visit with a treatment difference.

### Ethical considerations

The clinical trial was approved by the regional ethics committee in Stockholm. All patients signed a written consent form before entering the trial. The trial was conducted in accordance with the principles of the Declaration of Helsinki, and in compliance with International Conference on Harmonisation (ICH) guidelines and applicable local regulations. The clinical trial was approved by the Swedish Medicines Products Agency on EudraCT number: 2013-002126-23.

Due to the large drop-out rate in the Apovir group efficacy data for both the FAS (all patients with at least one efficacy recording after baseline) and the PPAS (all patients without a relevant major protocol deviation who attended all trial visits up to 1 month after end of treatment) have been analyzed statistically and are compared where relevant to justify the interpretations of the results.

## RESULTS

### Demographics and baseline characteristics

There were no major demographic differences between treatment groups as shown for the FAS in Table 1. Baseline disease characteristics (Table 1) as assessed by MMSE and ADAS-cog at baseline showed that patients in the Apovir group had slightly more progressed disease as compared to the patients in the placebo group whereas mean CDR-SB scores at baseline indicated no difference in disease severity.

**Table 1:** Demographic information and baseline characteristics, Full analysis set

	Apovir (*N* = 29)	Placebo (*N* = 33)
Age (y)
Mean (SD)	73.8 (5.5)	71.5 (5.9)
Min, Max	61, 85	61, 85
Sex n (%)
Female	14 (48.3%)	14 (42.4%)
Male	15 (51.7%)	19 (57.6%)
Ethnic origin n (%)
Caucasian	29 (100.0%)	33 (100.0%)
Disease characteristics at Baseline mean (SD)
MMSE score	23.4 (1.8)	23.9 (1.9)
ADAS-cog score	17.713 (5.9)	16.313 (5.5)
CDR-SB	4.21 (1.7)	4.12 (1.8)

MMSE, Mini-Mental State Examination; ADAS-cog, Alzheimer’s Disease Assessment Scale - cognitive; CDR-SB, Clinical Dementia Rating –sum of boxes.

**Fig. 1:**
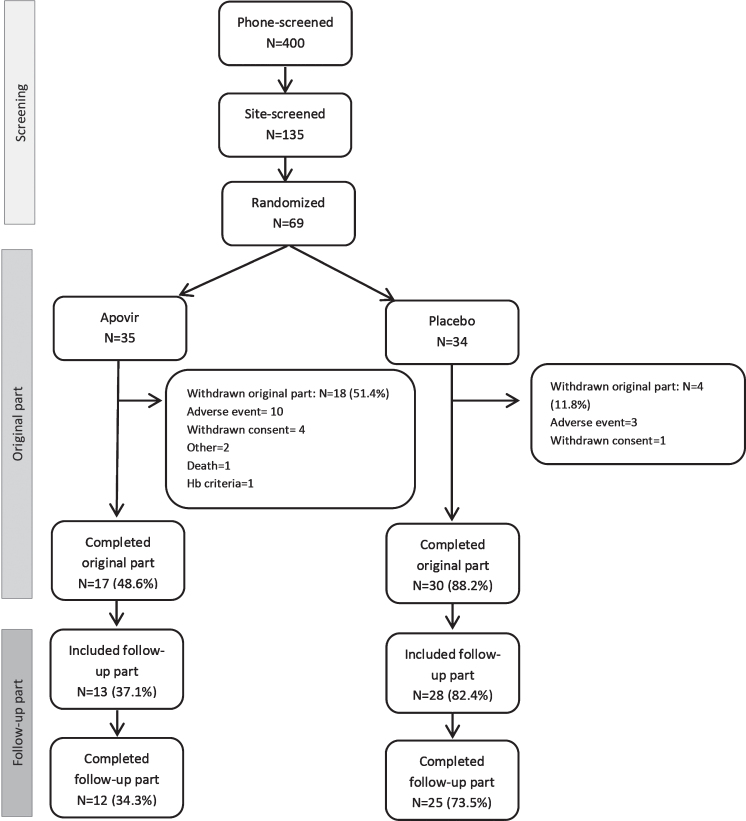
Disposition of patients and defined parts of the trial. The screening period comprised screening activities and visit up to randomization, the original part comprised the 9-month treatment period and 1-month follow-up visit, and the follow-up part comprise the 6- and 12-month follow-up visits.

Patient disposition data is shown in Fig. 1. In total, 47 patients (68.1%) completed the “original part” of the trial and 37 patients also completed the follow-up part of the trial. A large number of the patients in the Apovir group were prematurely withdrawn. A protocol amendment was made to allow patients to discontinue the ribavirin component of the treatment and continue in the trial with pleconaril alone. Three patients in the Apovir group and 1 patient in placebo discontinued ribavirin treatment prematurely during the trial. No separate analyses were made on this subgroup of patients.

In both treatment groups, the most common reason for premature discontinuation was AEs, reported by 12 (34.4%) patients in the Apovir group and 3 (8.8%) patients in the placebo group. The most frequent AE leading to withdrawal was fatigue reported for 3 patients and hallucination reported for 2 patients, with all other events reported only once. Additional details of AEs leading to withdrawal are shown in the Supplementary Table 1.

### Safety

Overall, the number of AEs reported in the trial was larger in the Apovir group compared to the placebo group, even with substantially shorter average exposure to treatment in the Apovir group (Supplementary Table 2). Although, a majority of the AEs were assessed as mild in both groups, a higher proportion of patients in the Apovir group reported AEs assessed as severe (17.1%) compared to the placebo group (5.9%). Further, in the Apovir group, 33.5%of the events (60 of 179 events) were assessed as either moderate or severe in intensity and in the placebo group the corresponding number was 10.9%(14 of 128 events). In addition, a higher proportion of the AEs reported in the Apovir group (41.3%; 74 of 179 events) were assessed as possibly or probably related to the study treatment, compared to the placebo group (21.9%; 28 of 128 events).

The most frequently reported AEs by treatment group are presented in Table 2. This table also displays the frequency of these events reported for ribavirin [79] and if the event has been reported previously for pleconaril [65, 67, 68].

**Table 2:** The most frequently reported adverse events according to MedDRA preferred term (≥10%in any treatment group), Safety analysis set and frequency of events reported for ribavirin and if event has been reported previously for pleconaril

Preferred term	Apovir (*N* = 35)		Placebo (*N* = 34)		AEs previously reported	
	Patients *n* (%)	Events	Patients *n* (%)	Events	Pleconaril	Ribavirin
Fatigue	12 (34.3%)	13	6 (17.6%)	7	No	≥10%
Hemoglobin decreased	16 (45.7%)	16	2 (5.9%)	2	No	≥10%
Headache	7 (20.0%)	11	9 (26.5%)	11	Yes	≥10%
Diarrhea	9 (25.7%)	11	4 (11.8%)	7	Yes	≥10%
Vomiting	5 (14.3%)	7	4 (11.8%)	4	No	1–10%
Nausea	6 (17.1%)	6	3 (8.8%)	4	Yes	≥10%
Weight decreased	8 (22.9%)	8	1 (2.9%)	1	No	1–10%
Back pain	4 (11.4%)	4	3 (8.8%)	3	No	1–10%
Fall	1 (2.9%)	1	5 (14.7%)	5	No	No
Syncope	4 (11.4%)	6	1 (2.9%)	1	No	1–10%
Rash	4 (11.4%)	4	1 (2.9%)	1	No	1–10%

*n*, number of patients for whom at least 1 event is reported. Percentages are based on the number of patients in the safety analysis set, broken down by treatment group.

Serious adverse events (SAEs) were more frequently reported in the Apovir group as shown in Supplementary Table 3. One death occurred in the Apovir group; this event was assessed as not related to the IMP treatment. In summary, safety data indicate tolerability was compromised but do not suggest patients were at risk for serious adverse consequences.

### ADAS-cog

The primary endpoint, change from baseline to 9 months in ADAS-cog total score, did not demonstrate a difference between groups *p* = 0.1809 (in PPAS *p* = 0.1197). However, within group changes in mean ADAS-cog total score showed a positive effect of Apovir (at 1-month follow-up) whereas in the placebo group there was a worsening (at 12-month follow-up), see Tables 2 and 3. The shape of the curves also indicate there may be a treatment effect as the curves deviated during treatment whereas during the follow-up period, the Apovir group turned and started to progress at a comparable rate to the placebo group.

A four-point change in ADAS-cog score was assessed as a clinically relevant change with an increase in score indicating a worsening and a decrease in score an improvement. An observed case analysis of the proportion of patients with clinically relevant change showed a difference between treatment groups at 10 months (*p* = 0.0329). Interestingly, the number of patients with a clinically relevant improvement in the Apovir group was larger at 6 and 10 months and at 12-months follow-up compared to placebo despite the smaller number of patients in the Apovir group. Also, the proportion of patients with a clinically relevant worsening, was larger in the placebo group at all time-points (Table 4). Results in PPAS showed a statistically significant difference between groups at 6 months (*p* = 0.0489).

### CDR-SB

There was no difference in CDR-SB between treatment groups at either 9 months or 1-month follow-up (*p* = 0.5251 and *p* = 0.4329, respectively). However, data showed a worsening (increase in score) from baseline to 9 months in the placebo group (*p* = 0.0029; 1.36±2.48), whereas the worsening at 1-month follow-up (1.24±3.04) was not confirmed (*p* = 0.0712). In the Apovir group, the worsening was less pronounced than in the placebo group and the effect was not confirmed at 9 months or at 1 month after end of treatment (0.50±1.98 *p* = 0.2526; 0.50±2.03 *p* = 0.6088).

### MMSE

Descriptive data on observed cases showed a decrease in MMSE score from baseline at all time-points in both treatment groups during the trial. Mean baseline score per treatment group is provided in Table 1. In the FAS, the largest decrease compared to baseline was –1.7 in the Apovir group, observed at 9 months and –1.7 in the placebo group, observed at 1-month follow-up. Results were in favor of placebo at 6 and 9 months and in favor of Apovir at 1-month follow-up. The largest difference between groups was observed at 1-month follow-up when the change from baseline was –0.2 and –1.7 in Apovir and placebo, respectively. There was no clear trend for a difference between groups.

### Plasma and cerebrospinal fluid IMP concentrations

IMP plasma concentrations were assessed before the morning dose at all visits from 1 month to 9 months, and CSF concentrations of IMP were assessed at the 9-month visit. The highest mean plasma concentration of ribavirin, 6.84μmol/L was observed at the 3-month visit. Ribavirin plasma concentrations were within the expected range based on previous reports [80, 81] and considering the general dose reduction in the trial. Plasma concentration of pleconaril increased throughout the trial and there was an unexpected delay in steady state. The mean plasma concentration level increased from 1,362.9 ng/ml at 1 month to 2,315.6 ng/ml at 9 months. Additional analyses confirmed that this increase over time was not due to the drop-out of patients (not shown). Both pleconaril and ribavirin were present in the CSF, with mean concentrations at 9 months of 6.58 ng/ml and 3.61μmol/L respectively for pleconaril and ribavirin. The ratio of ribavirin concentrations between CSF and plasma at 9 months was 89.2%(*n* = 9) for pleconaril the ratio is not provided as it would compare free concentration in CSF versus total amount (free and plasma protein bound) in plasma.

### Association IMP plasma and CSF concentrations and change in ADAS-cog

The associations between pleconaril and ribavirin plasma and CSF concentrations and change in ADAS-cog score from baseline to 9 months showed a stronger correlation between change in ADAS-cog and pleconaril (plasma = –0.42, CSF = –0.15) compared to ribavirin (plasma = –0.05, CSF = 0.09).

### CSF biomarkers Aβ, tau, and p-Tau

CSF biomarkers were assessed at baseline and at the end of the treatment period, at 9 months. Baseline results and change from baseline to 9 months are shown in Table 5. *Posthoc* statistical tests showed no statistically significant difference in change between the two treatment groups for any of the biomarkers assessed. Changes within groups using the PPAS showed a statistically significant decrease in Aβ^42^ change from baseline to 9 months in the Apovir group (*p* = 0.0330), but no statistically significant change in the placebo group. Tau and p-Tau changes were not statistically significantly changed in either placebo or Apovir. When all patients with both baseline and 9-month data were included in the analysis, results were comparable to the results in the PPAS. The correlation between change in ADAS-cog and Aβ _42_ from baseline to 9 months in placebo was –0.09 and in Apovir –0.31.

**Table 5:** CSF biomarkers at baseline and change from baseline to 9 months, Per protocol analysis set

	Baseline Aβ_42_	Change to 9 Months Aβ_42_	Baseline Tau	Changes to 9 Months Tau	Baseline *p*-Tau	Changes to 9 Months *p*-Tau
Apovir n/nmiss	9/1	9/1	9/1	9/1	9/1	9/1
Mean (SD)	568.8 (129.0)	–70.4 (80.8)^ *^	667.6 (331.3)	–24.1 (155.9)	97.0 (64.9)	–18.6 (43.9)
Median	530.0	–75.0	549.0	6.0	82.0	–5.0
Placebo	22/3	17/8	22/3	17/8	22/3	17/8
Mean (SD)	573.5 (145.3)	2.9 (86.3)	788.3 (317.7)	–25.6 (142.8)	102.9 (35.9)	–8.9 (13.1)
Median	576.0	–7.0	646.5	0.0	95.0	–10.0

^ *^Paired *T*-test *p* = 0.0330 within group change from baseline to 9 months. Remaining tests within group and between group changes not significant.

### Interim analysis

A sample size re-calculation was performed as planned when 30 patients had performed the 6-month visit. The results confirmed the planned sample size was and resulted in the conclusion that no additional patients were to be included.

### Effect of drop-out

Several analyses were made to assess if the uneven drop-out rate has affected the results. In summary, the results from these analyses suggest the effect observed cannot solely be explained by the drop-out. 1) Results on effect size estimates for ADAS-cog at 9 months, using z-score imputation, show that following 3 iterations there is still an effect in the Apovir group indicating a complete halt of disease progression (see Supplementary Table 4). 2) Evaluation of ADAS-cog and CDR-SB results based on patients included in the PPAS or the FAS only where patients in FAS were considered as drop-outs was made to assess if the reason for the signs of effect in the trial could be explained by a difference in disease progression in drop-outs compared to non-drop outs. Results in Supplementary Table 5 showed no statistically significant difference between drop-outs and non-drop outs within either the Apovir or the placebo group during the original part of the trial suggesting that reason for drop-out may be related to lack of efficacy in addition to safety problems. 3) Removing matched ranked placebo patients to explore the effect and potential bias of the results due to the high drop-out rate in the Apovir group. The patients were ranked separately within each treatment group at baseline and were then matched 1 : 1 with the same ranking patient from the other group. Patients who dropped out of the Apovir group had their matched placebo subject dropped from the analysis at the time of Apovir patient drop-out. Effect size of original scores and z-score imputed scores were analyzed for ADAS-cog, CDR-SB, ADCOMS, and MMSE at 9 months (see Supplementary Table 6). ADAS-cog, ADCOMS, and CDR-SB data indicate a treatment effect of Apovir whereas MMSE results were in favor of placebo. 4) Removing the patients in the placebo group; with the worst (highest) ADAS-cog score at baseline, and with the largest worsening (increase in score) in ADAS-cog at 9 months corresponding to the number of patients who dropped out of the Apovir group (see Supplementary Table 7). Results show that the difference between treatment groups remained when the worst placebo patients at baseline were removed. Only when the worst responders in the placebo group were removed (a worst-case analysis), was there no notable difference between the two treatment groups. 5) MMRM analysis of ADAS-cog at 9 months showed a change from baseline of 0.743 (±1.537) in the Apovir group and 2.018 (±1.315) in the placebo group. There was no statistically significant change either between groups (*p* = 0.5376) or within groups (*p* = 0.6294 in Apovir and 0.1265 in placebo). Due to the large drop-out rate in the Apovir group, it could be presumed that drop-out of patients with concurrent diseases known to affect AD could affect the results. The most commonly reported concurrent diseases in the trial were hypertension (40.0%/41.2%; Apovir/placebo), hyperlipidemia (25.7%/32.4%; Apovir/placebo), and depression (28.6%/23.5%; Apovir/placebo). No formal analyses were conducted on effect of drop-out in relation to these concurrent diseases but judged from descriptive data, there was no clinically relevant over representation in drop-out patients with these diseases (data not shown).

## DISCUSSION

The two primary aims of this trial were to assess the safety of Apovir and the efficacy of Apovir related to AD progression. The primary efficacy endpoint was not met and due to a large drop-out rate the trial is judged as inconclusive. However, there were indications of a potential positive treatment effect of Apovir. Although these indications of effect need to be verified in future trials, we observed a difference in clinically relevant change between treatment groups assessed by ADAS-cog and defined as a 4-point change from baseline, a cognitive improvement in the Apovir group as assessed by ADAS-cog and a worsening in the placebo group as assessed by CDR-SB. Further, the difference in the shape of the ADAS-cog curves over time provide additional indications of a potential treatment effect; with a continuous worsening in the placebo group throughout the trial whereas in the Apovir group, the curve indicates an improvement during the treatment period and a worsening after end of treatment comparable to that of placebo. Although these results are encouraging, the MMSE showed slightly better scores in placebo at months 6 and 9, and slightly better scores in Apovir at 1-month follow-up indicating that MMSE data does not provide support to the effects seen in ADAS-cog. MMSE is generally regarded as a less sensitive tool to assess disease progression in AD compared to ADAS-cog and CDR, still this lack of consistency between data further point at the need for caution when interpreting the efficacy results of this trial.

Apovir was judged to be generally safe but the tolerability was compromised as evident from both a high frequency of AEs and a high drop-out rate. During the analyses of the trial data major efforts were made to delineate 1) which part of Apovir caused the negative effects (AEs and drop-out), 2) the effect of drop-out on the interpretations of the efficacy results, and 3) how the two respective components of Apovir, pleconaril and ribavirin, contributed to the potential positive treatment effect. It shall be emphasized that the trial was not designed to assess the contributions of the respective components of Apovir. The attempts described below to identify any such signs were made to guide the decisions on the future development and should be considered exploratory.

### Reason behind negative effects of Apovir

In previous trials with pleconaril alone, tolerability has been favorable although gastrointestinal symptoms including nausea and diarrhea as well as headache have been reported. In this trial, both nausea and diarrheas were more frequently reported in the Apovir group and may thus be related to the pleconaril whereas headache was more frequent in the placebo group. The treatment periods in earlier trials with pleconaril have been relatively short, up to 6 weeks, compared to the considerably longer 9 months in this trial. Long treatment periods have thus not been studied previously and it is possible that the AE profile during long term treatment is changed for pleconaril. It is also possible that the delay in plasma concentration steady state for pleconaril observed in this trial contributed to the increased frequency of AEs, but this is not supported by the temporal association of the AEs (data not shown). The pharmacokinetics of long-term pleconaril treatment will need to be confirmed during the continued development, but available data does not indicate that the delay in plasma concentration steady state has contributed to the adverse effects. Based on our evaluations, we believe a major part of the tolerability issues in this trial were caused by the ribavirin part of Apovir primarily because the well-known side effects of ribavirin correlated very closely with the AEs reported in the trial (as shown in Table 2). In fact, all of the 10 AE terms that were reported in at least 10%of the patients in the Apovir group, are listed as reported in≥1%ribavirin users [79], and the two most frequently reported AEs in the trial, decreased levels of hemoglobin and fatigue, are both key features during ribavirin treatment. The clinical experience from ribavirin is extensive in the adult population but the experience from treatment of elderly patients is limited. However, similar to the results reported in this trial, another study has also reported a higher treatment discontinuation rate and more AEs in a geriatric population treated with ribavirin compared to non-geriatric adults [82]. Thus, even though we had attempted to limit the risk of ribavirin related AEs by selecting a dose which is about half of the commonly used dose of ribavirin, the tolerability for Apovir appears to have been compromised by ribavirin.

### Effect of drop-out

The tolerability issues in the trial lead to the large drop-out rate which made the efficacy results difficult to interpret and resulted in an inconclusive trial. Several *post-hoc* analyses were conducted in an attempt to address the effect of drop-out. When the drop-out effect is considered by different methods, as reported above, results indicate that part of the effect is lost but also that drop-out cannot explain the entire effect observed, supporting additional trials to confirm if there is an effect. It is worth noting that the number of patients with a clinically meaningful improvement in ADAS-cog is larger in the Apovir group compared to placebo at several time-points despite having considerably fewer patients in this treatment group due to drop-out. This observation is judged as a sign of a possible treatment effect.

### Contributions from pleconaril and ribavirin

The trial was not designed to distinguish between the effect of the two components of Apovir but in view of the tolerability issues encountered it became important to explore how ribavirin and pleconaril each might have contributed to the potential effect observed. Our main rational for including ribavirin was to prevent the development of viral resistance towards pleconaril. However, ribavirin also has effect on several other viruses that have been connected to AD and further it has immunomodulatory effects that may also have contributed with an effect in the trial. There are no data to discriminate between the effect of the two drugs but the associations between change in ADAS-cog from baseline to 9 months and plasma and CSF concentrations of pleconaril were stronger than for ribavirin indicating that pleconaril may be more important for the effect than ribavirin. If we are to treat a persistent infection in the brain it would be important that the drug gains access to the brain. Only a very low free concentration of pleconaril was found in the CSF, this result was expected based on both the plasma protein binding properties (99.9%) of the compound and on previous tissue distribution studies in rats which showed that [^14^C]-pleconaril was extensively distributed into most tissues, and further that the concentrations of drug-derived radioactivity in the brain, sciatic nerve, and spinal cord remained substantially higher than those in plasma and cerebrospinal suggesting a partitioning into tissues of the CNS. The overall distribution of [^14^C]-pleconaril, and the prolonged association of radioactivity with lipid-rich tissues, is consistent with its lipophilic nature. For ribavirin which does not bind to plasma proteins, the ratio of drug found in CSF compared to plasma was high and is consistent with its water-soluble properties whereas high concentrations would not be expected in the brain. High CSF concentration does not imply high brain concentration. Ferrara et al. reported in a distribution study that in both rats and monkeys treated with a single dose of [^14^C]ribavirin, the brain was the tissue with the lowest concentration of radioactivity of all tissues evaluated, only about 1%of a single dose of ribavirin given intravenously to Rhesus monkeys was detected in the brain [83]. Therefore, neither the pleconaril nor the ribavirin concentrations in the CSF versus plasma predict the amount of drug found in the brain and these results combine to suggest that pleconaril is more likely to exert antiviral effects in the brain compared to ribavirin. Information in the literature shows that the effect of ribavirin on EV should be regarded as modest based on the antiviral efficacy in clinically relevant doses [58, 84] and the doses used in this trial were lower than those commonly used. Thus, we judge that low dose ribavirin is not likely to have contributed considerably to an EV antiviral effect in this trial or to prevent the development of viral resistance towards pleconaril since it is not expected to have reached sufficient concentration to exert such effect. Ribavirin has received regulatory approval are hepatitis C and for RSV infections in children. However, use of ribavirin as monotherapy for hepatitis C is not approved and it has been shown that oral formulations of ribavirin alone has no beneficial effect on treatment of chronic hepatitis C or on virologic response in suppressing the hepatitis C virus, compared to placebo [85]. Also ribavirin treatment in children with RSV has recently been questioned since a systematic analysis failed to demonstrate therapeutic value from the treatment. For this reason, at least in some countries, treatment with ribavirin for RSV in children has practically ceased to be used [86] although it is still used at high doses to treat RSV off-label in adults. The lack of evidence for a clear antiviral effect from ribavirin in approved indications even at doses double the doses used in the herein reported trial supports our view that it is unlikely that ribavirin in this trial has contributed with an antiviral effect. Since HSV has been implicated in AD and since ribavirin may have some anti-HSV effects it should be emphasized that this trial was not aimed at studying potential antiviral effect of ribavirin on HSV. It is still possible that ribavirin could have contributed by targeting inflammatory processes by its immunomodulatory effects.

In combination with the indications of potential effect observed, it is interesting that the CSF biomarker levels showed a decrease in Aβ_42_ in the Apovir group during the treatment period. Such effect has been observed also with other agents, rifampicin and caffeine, and has been suggested to be at least part of the mechanism behind their potential protective effect against AD [87]. It is possible that this decrease in Aβ_42_ reflects a reduced production of Aβ in the brain and further that this could be due to a decrease in brain infection. If Apovir, which does not directly target Aβ, still has an effect on Aβ levels and if this is part of the mechanism by which Apovir exerts its effects in AD, these results would argue for the importance of finding the balance of Aβ between a protective defense protein and an inflammatory stimulating agent with toxic properties. In some ways this supports the amyloid hypothesis, where viral infection (among other possible triggers) could lead to over production of amyloid at a key time in life, when amyloid clearance is reduced. Antiviral therapy reduces the need for innate amyloid production, thereby reducing the levels of what is clearly a contributing factor to the clinical AD syndrome.

No testing for *APOE4* was performed, which is a potential weakness of the study. Patients with AD have a high presence of *APOE4* and this has in many clinical trials with anti-AD drugs been shown to be of importance. Thus, we could not study the potential effect of this gene influence on the treatment.

In summary, the trial efficacy results suggest that there might be a treatment effect with Apovir whereas the safety results indicate a need for improved tolerability of the treatment. In view of our hypothesis, that EV is involved in the development and or progression of AD, we aimed to test this hypothesis in a larger proof of concept trial evaluating the effect of pleconaril alone. However, there was no evidence of EV infection in the patients participating in the trial since this was not assessed, but as pointed out above, patients were expected to encounter EV infections during the trial treatment period. We also need to point out that the results appear consistent with the hypothesis that partial inhibition of another virus type sensitive to ribavirin might have contributed to the results although we deem that less likely, and that ribavirin’s immunomodulatory effects might underlie the signs of clinical benefits reported in this trial. New trials evaluating the effect of pleconaril are needed. Such trials together with the two ongoing clinical trials evaluating the effect of the HSV medicine Valacyclovir on AD, may together contribute to a better understanding of a potential viral contribution from EV and HSV in AD and the possibility of slowing the disease progression with antiviral treatments.

## Supplementary Material

Supplementary Material
