# Supplementary material for: Potential Virus Involvement in Alzheimer’s Disease: Results from a Phase IIa Trial Evaluating Apovir, an Antiviral Drug Combination
Source: J Alzheimers Dis Rep. 2021 May 28;5(1):413–31. doi: 10.3233/ADR-210301 (PMC8203284; doi:10.3233/ADR-210301)
Supplement: Supplementary Material [file adr-5-adr210301-s001.pdf]

Supplementary Material

Potential Virus Involvement in Alzheimer's Disease: Results from a Phase IIa Trial Evaluating Apovir, an Antiviral Drug Combination

Supplementary Table 1. Adverse events leading to withdrawal

AE leading to withdrawal	Apovir Patients n	Placebo Patients n
Arrhythmia	1*	0
Bladder neoplasm	1	0
Blister	0	1*
Breast cancer	1	0
Diarrhea	1	0
Fatigue	3*	0
Hallucination	1*	1*
Lung neoplasm	1	0
Nausea	0	1*
Pneumonia	1	0

*Causality assessed as possibly of probably related to treatment

Supplementary Table 1. Number of adverse events and relation to exposure to investigational products

**Total number of AEs**	**Apovir N=35 179**		**Placebo N=34 128**	
	Pleconaril	Ribavirin	Placebo pleconaril	Placebo ribavirin
Exposure days/patient, Mean* (SD)	193 (94)	172 (102)	251 (59)	245 (70)
Exposure patient days (y)*	6755 (18.5)	6020 (16.5)	8534 (23.4)	8330 (22.8)
AEs per patient year	9.7	10.8	5.5	5.6

*The difference in days of exposure between pleconaril and ribavirin is due to premature withdrawal of ribavirin which was allowed according to the protocol if tolerability was compromised, and patients wanted to end the trial prematurely or if adverse events prevented reintroduction following a dose interruption

Supplementary Table 3. Serious adverse events by term and treatment group

SAE term<br>System organ class/Preferred term	Apovir	Placebo
**Total number of SAEs**	**12**	**3**
**Infections and infestations**	**2**	**0**
Pneumonia	1	0
Urinary tract infection	1	0
**Neoplasms benign, malignant, and unspecified (including cysts and polyps)**	**3**	**0**
Bladder neoplasm	1	0
Breast cancer	1	0
Lung neoplasm	1	0
**Blood and lymphatic system disorders**	**1**	**0**
Anemia	1	0
**Nervous system disorders**	**1**	**0**
Depressed level of consciousness	1	0
**Cardiac disorders**	**1**	**1**
Atrial fibrillation	1	0
Tachycardia	0	1
**Gastrointestinal disorders**	**1**	**0**
Abdominal discomfort	1	0
**Musculoskeletal and connective tissue disorders**	**0**	**1**
Musculoskeletal pain	0	1
**Renal and urinary disorders**	**1**	**0**
Hematuria	1	0
**General disorders and administration site conditions**	**2**	**1**
Chest pain	0	1
General physical health deterioration	1	0
Medical device complication	1	0

Supplementary Table 2. ADAS-cog effect size estimates for additional iterations of Z-score imputation

	Observed cases	First iteration z-score	Second iteration z-score	Third iteration z-score
Effect at 9 months	169%	126%	110%	101%

Analyses based on observed cases are impacted by drop-out biases and should be interpreted cautiously.

The meaning of effect size and z-score imputation are clarified in the statistical section.

All analyses in this table included data from discontinuation visits that occurred close to the scheduled visit date, although these data were not included in the primary analysis. The effect size for observed cases will not match the primary analysis due to this difference.

Supplementary Table 3. Comparison of drop-outs (FAS only) and non drop-outs (PPAS) by treatment. Change from baseline in ADAS-cog and CDR-SB

			ADAS-cog			CDR-SB	
**Treatment**	**Drop-out**	**Statistics**	**6M**	**9M**	**1M FU**	**9M**	**1M FU**
Apovir	PPAS	N/Nmissing	9/1	10/0	10/0	10/0	10/0
		Min, Max	-9.67, 8.67	-11.33, 3.33	-5.33, 2.33	-4.00, 2.50	-2.00, 4.50
		Median	-4.00	-3.00	-4.00	0.00	-0.25
		Mean (SD)	-3.93 (5.84)	-2.80 (3.94)	-2.70 (2.92)	-0.20 (1.89)	0.05 (1.83)
	FAS only	N/Nmissing	11/8	7/12	6/13	7/12	6/13
		Min, Max	-6.67, 9.67	-9.33, 5.00	-7.67, 5.00	-1.50, 3.50	-2.00, 4.00
		Median	-1.33	2.00	-4.67	1.50	1.25
		Mean (SD)	-0.15 (4.90)	-0.90 (5.37)	-3.17 (4.72)	1.50 (1.76)	1.25 (2.30)
	p		0.0912	0.3743	0.4737	0.0887	0.2705
Placebo	PPAS	N/Nmissing	24/1	25/0	25/0	24/1	25/0
		Min, Max	-12.00, 6.33	-6.00, 16.67	-9.00, 18.00	-2.00, 4.00	-2.50, 5.00
		Median	-1.00	-1.33	-1.67	1.00	0.50
		Mean (SD)	-0.82 (4.11)	0.09 (4.84)	-0.53 (5.72)	0.83 (1.49)	0.74 (2.09)
	FAS only	N/Nmissing	6/2	6/2	5/3	4/4	4/4
		Min, Max	-3.33, 21.67	-4.67, 40.33	-3.67, 29.67	-1.00, 9.00	-3.00, 11.00
		Median	1.33	2.83	0.33	5.00	4.75
		Mean (SD)	4.94 (9.51)	9.83 (17.72)	8.73 (14.57)	4.50 (4.80)	4.38 (6.02)
	p		0.1957	0.3323	0.1480	0.1868	0.1915

Analyses based on observed cases are impacted by drop-out biases and should be interpreted cautiously

Supplementary Table 4. Estimates of effect size at 9 months for original data and z-score imputed data with placebo matched rank subjects dropped at the same time of active patient drop-out

	Original scores	Z-score imputed scores
ADAS-cog	175%	101%
ADCOMS	48%	47%
CDR-SB	51%	51%
MMSE	77%*	53%*

*Effect is in favor of placebo.

The meaning of effect size and z-score imputation are clarified in the statistical section. Although the numbers in this table are based on an analysis that removes placebo subjects with matched ranks to the active dropout subjects at baseline, the effect size at 9 months for the z-score imputed values turns out to match (within rounding error) the effect size at 9 months (101% from Supplementary Table 4) for the z-score imputed values without removing the placebo matched subjects. This is because the placebo subjects that were removed had similar decline to the remaining placebo subjects after doing the z-score imputation.

Supplementary Table 5. Change in ADAS-cog score from baseline after removing worst patients from the placebo group

Change in ADAS-cog score from baseline	6 months	9 months	1 month FU
Apovir	-0.379	-1.702	-2.188
Placebo patients dropped based on:			
Worst baseline ADAS-cog	0.873	2.546	1.856
Worst response in ADAS-cog at 9 months	0.742	-2.146	-2.194
